# Authenticity and geographic origin of global honeys determined using carbon isotope ratios and trace elements

**DOI:** 10.1038/s41598-018-32764-w

**Published:** 2018-10-02

**Authors:** Xiaoteng Zhou, Mark Patrick Taylor, Helen Salouros, Shiva Prasad

**Affiliations:** 1 Department of Environmental Sciences, Faculty of Science and Engineering, Macquarie University, North Ryde, Sydney, New South Wales 2109 Australia; 2 Energy and Environmental Contaminants Research Centre, Macquarie University, North Ryde, Sydney, New South Wales 2109 Australia; 3 Australian Forensic Drug Laboratory, National Measurement Institute, North Ryde, Sydney, New South Wales 2113 Australia; 4 Analytical Service Branch, National Measurement Institute, North Ryde, Sydney, New South Wales 2113 Australia

## Abstract

Honey is the world’s third most adulterated food. The addition of cane sugar or corn syrup and the mislabelling of geographic origin are common fraudulent practices in honey markets. This study examined 100 honey samples from Australia (mainland and Tasmania) along with 18 other countries covering Africa, Asia, Europe, North America and Oceania. Carbon isotopic analyses of honey and protein showed that 27% of commercial honey samples tested were of questionable authenticity. The remaining 69 authentic samples were subject to trace element analysis for geographic determination. One-way ANOVA analysis showed a statistical difference (*p* < 0.05) in trace element concentrations of honey from Australian regions and different continents. Principal component analysis (PCA) and canonical discriminant analysis (CDA) coupled with C5.0 classification modelling of honey carbon isotopes and trace element concentrations showed distinct clusters according to their geographic origin. The C5.0 model revealed trace elements Sr, P, Mn and K can be used to differentiate honey according to its geographic origin. The findings show the common and prevalent issues of honey authenticity and the mislabelling of its geographic origin can be identified using a combination of stable carbon isotopes and trace element concentrations.

## Introduction

Genuine pure honey is classified as a natural product produced entirely by bees. Formally, it is defined as “…the natural sweet substance, produced by honey bees from the nectar of plants or from secretions of living parts of plants or excretions of plant-sucking insects on the living parts of plants, which the bees collect, transform by combining with specific substances of their own, deposit, dehydrate, store and leave in honeycombs to ripen and mature”^1,2^.

Honey is a natural sweetener containing sugars and small quantities of minerals, vitamins, proteins, fatty acids, amino acids. Its nutritious components make honey suitable for a wide range of applications in the food industry, such as cooking, baking, desserts and beverages. Honey also has medicinal properties due to its antioxidant and antimicrobial activities, resulting in specific types of honey, for example New Zealand manuka honey^3^, having significant commercial value.

Manuka honey is New Zealand’s most iconic honey and commands a premium price due to its claimed health-related benefits^3^. The annual production of manuka honey in New Zealand is only 1,700 tons^4^. However, it is estimated that as much as 10,000 tons of New Zealand manuka honey is sold globally each year^4^. The global market value of honey was worth an estimated US$6.6 billion in 2015^5^.

There was an escalation in the practice of adulterating honey in world markets from the 1970s following the introduction of high fructose corn syrup^6^. Corn syrup and sugar cane, both a cheaper sugar source than honey, are added commonly to honey to increase product volume, which is then traded as a genuine pure honey^7^. Corn syrup and sugar cane are sourced from C-4 plants with the produced sugars reflecting their original carbon isotopic composition. By contrast, bees collect nectar and pollen for honey production primarily from the flowers of C-3 plants, and to a lesser extent from the flowers of C-4 plants^7^. Sugar syrups produced by the C-4 metabolic pathway exhibit a ^13^C/^12^C ratio (expressed as δ^13^C) that differs from sugars derived from the C-3 metabolic pathway (−10‰ to −20‰ for C-4 plants, and −22‰ to −33‰ for C-3 plants)^8,9^.

The δ^13^C value in pure honey is relatively uniform, with δ^13^C values >−23.5‰ classified as adulterated with lower δ^13^C syrups according to the AOAC (Association of Official Analytical Chemists) Official Method 978.17^10^. Also, honey is considered to be adulterated if it contains C-4 sugars >7%, according to the following equation (1)^11^:

1 $$C-4\,Sugars,\, \% =\frac{{{\rm{\delta }}}^{13}{C}_{protein}-{{\rm{\delta }}}^{13}{C}_{honey}}{{{\rm{\delta }}}^{13}{C}_{protein}-(\,-\,9.7)}\times 100$$

where δ^13^*C*_*protein*_ and δ^13^*C*_*honey*_ are δ^13^C values (‰), for protein and honey, respectively, and −9.7 is the average δ^13^C value for corn syrup (‰).

In addition, honey with C-4 sugars <−7% can also be classified as adulterated^12^. Although the analysis of δ^13^C in honey and calculation of the proportion of C-4 sugars is useful for detecting adulteration by the addition of syrups, false positive results may occur if honey is produced naturally from C-4 plants^13^. High values of the non-peroxide activity (NPA >10+) and methylglyoxal (MGO >250 mg/kg) in New Zealand manuka honey can increase the possibilities of false positive results^14^. This limitation of the carbon isotope method can potentially be addressed via a comparison of the carbon isotope ratios of bulk honey to honey protein^7^. The protein acts as an internal control, given that its δ^13^C value is unaffected by adulteration^9^. By contrast, the δ^13^C value of honey changes with addition of sugar causing a difference to occur between δ^13^C values in honey and protein^15^. A difference >1‰ in δ^13^C values (δ^13^C_honey-protein_ expressed as δ^13^C_h-p_) is considered to indicate that the protein and the bulk honey have different origins^16^ resulting in such honeys being classified as adulterated^8,15,17–20^. Consequently, stable carbon isotope ratio analysis has been used for decades as an analytical tool to detect honeys that have been adulterated with C-4 sugars^13,17,21^.

As well as the addition of sugar to honey, mislabelling of the geographic origin of food is a growing worldwide problem^22–24^ including in the honey market^25^. According to the Codex Alimentarius Commission Standards^2^ and European Commission^1^, the geographic origin of honey should be the same as the area declared on its label. Deliberate mislabelling of honey origin has been reported frequently in the media^26–30^. This not only compromises the confidence of customers with respect to the authenticity of certified regional products, but raises health and safety concerns as blended honey of unknown origin has been known to contain antibiotics, toxins, irradiated pollen or even alkaloids with the potential to cause organ damage^31^.

Although honey trace element profiles have previously been related to their geographic source at a regional scale^32–35^, no study has investigated if samples can be separated at a broader continental level to authenticate their origin. Further, while trace element analysis has been used to confirm the authenticity of honey labelled with place of origin in Spain^36^, Turkey^32^, Argentina^33^, Slovenia^37^, Brazil^34^, Italy^35^ and Romania^38^, no study has evaluated Australian honey with the aim of developing a method to distinguish its honey from international products. Australia is the world’s 4^th^ largest exporter of honey^39^. The Australian honey industry, including pollination services was estimated to be worth at least AUD$4–6 billion per annum in 2008^40^. Australian honey is characterised as safe and high quality since it is produced in a “clean and green” country^41^ with one of the world’s most rigorous apicultural management systems. However, recent scandals in which the “Australian product” logo was used falsely on products purporting to be Australian honeys^26,27^ has raised public concern about the stated authenticity of honey origin and quality.

This study uses stable carbon isotope ratio analysis to investigate the authenticity of honey from mainland Australia (n = 29), Tasmania (n = 9) and 54 honeys from 18 other countries across five continents. The non-Australian samples were derived from Africa (n = 1), Asia (n = 21), Europe (n = 21), North America (n = 9) and Oceania (n = 2 from New Zealand), with three samples of an unknown origin. Trace element analyses of Australian and international honeys were undertaken with the aim of ascertaining if there were separate and distinct concentrations according to regions and continents and if this approach could be used to verify geographic origin.

## Results

Five raw honey samples were collected from Sydney (New South Wales) and Calliope (Queensland) hives, to: (a) demonstrate that they matched the AOAC criteria for δ^13^C in pure honey, its protein and C-4 sugar and (b) benchmark δ^13^C and C-4 sugar values measured in 95 commercial honeys.

The 95 commercial honey samples from 19 countries were assessed according to the following criteria for potential adulteration: δ^13^C_honey_ > −23.5‰^10^; C-4 sugar >7%^11^ and <−7%^12^; and δ^13^C_h-p_ >1‰^8,15,17–20^. Honey samples that passed the C-4 sugar criteria were assumed to be authentic given that C-4 sugar is the only adulterant that has an official detection method^42^.

Honey samples that passed the C-4 criteria were subjected to multi trace element analysis. The 16 trace element concentrations coupled with δ^13^C values in bulk honey and its protein were used to determine the geographic origin of the honey using PCA and CDA statistical analysis. In addition, C5.0 classification modelling, a commonly used tool in data mining, was used to evaluate δ^13^C values and trace element concentrations to ascertain if samples could be separated according to their geographic origin. The full dataset for δ^13^C in honey and protein is provided in Supplementary Table S1. Trace element concentrations of Al, Ba, B, Ca, Cu, Fe, Mg, Mn, Ni, P, K, Rb, Na, Sr, Sn and Zn are provided in Supplementary Table S2.

### Carbon isotope analyses

The δ^13^C values in raw honey samples (n = 5) were <−23.5‰^10^, and their C-4 sugars were <7%^11^ (Supplementary Table S1). Also, the difference in δ^13^C values between the bulk honey and its protein met the criterion used widely, that being ≤1‰ (δ^13^C_h-p_ ranged from −0.61‰ to −0.08‰) (Supplementary Table S1), corresponding to <7% added C-4 sugars^8,15,17–20^. The most recent AOAC 998.12^11^ sets the upper acceptable limit for C-4 plant sugars in honey at ≤7%. The linear relationship for raw honey and its protein was: δ^13^C_protein_ = 0.655 × δ^13^C_honey_ − 8.67 (R^2^ = 0.932, *p* = 0.008).

Commercial honey samples (n = 95) had δ^13^C values for honey and protein that ranged from −27.91 to −13.35‰ and −27.78 to −22.30‰, respectively. Seventeen of these commercial honey samples (17.9%) were found to be potentially adulterated (Table 1) according to the AOAC 998.12^11^, which states that honey samples are considered to be adulterated if they contain C-4 sugars >7%.

**Table 1:** Data for δ^13^C_honey_ (‰), δ^13^C_protein_ (‰), δ^13^C_h-p_ (‰), C-4 sugar (%) and detection criteria in adulterated commercial honey samples from mainland Australia (M-AUS, n = 5), Tasmania (TAS, n = 2), Asia (AS, n = 11), Europe (EU, n = 6) and two Oceanic samples (OA) from New Zealand.

Sample No.	Countries	δ^13^C_honey_ (‰) Criterion^10^: <−23.5^a^	δ^13^C_protein_ (‰)	δ^13^C_h-p_ (‰) Criterion^8,15,17–20^: ≤1^b^	C-4 sugar (%) Criteria^11,12^: ≤7^c^ or >−7^d^
M-AUS-25	Australia	−26.40 ± 0.05	−25.21 ± 0.06	−1.19	−***7***.***66***^***d***^
M-AUS-26	Australia	−26.74 ± 0.06	−25.62 ± 0.14	−1.12	−***7***.***02***^***d***^
M-AUS-27	Australia	−26.66 ± 0.04	−25.10 ± 0.18	−1.56	−***10***.***14***^***d***^
M-AUS-28	Australia	−25.33 ± 0.09	−26.80 ± 0.12	***1***.***48***^***b***^	***8***.***63***^***c***^
M-AUS-29	Australia	−24.37 ± 0.09	−25.44 ± 0.11	***1***.***07***^***b***^	6.80
TAS-8	Australia	−23.68 ± 0.08	−25.17 ± 0.09	***1***.***49***^***b***^	***9***.***64***^***c***^
TAS-9	Australia	−23.84 ± 0.06	−25.22 ± 0.26	***1***.***38***^***b***^	***8***.***91***^***c***^
AS-36	China	−25.25 ± 0.06	−23.70 ± 0.09	−1.55	−***11***.***04***^***d***^
AS-37	China	−***15***.***52***^***a***^ ± ***0***.***04***	−23.71 ± 0.10	***8***.***18***^***b***^	***58***.***43***^***c***^
AS-38	India	−24.68 ± 0.11	−27.02 ± 0.12	***2***.***34***^***b***^	***13***.***52***^***c***^
AS-39	Indonesia	−***21***.***58***^***a***^ ± ***0***.***06***	−26.05 ± 0.17	***4***.***47***^***b***^	***27***.***35***^***c***^
AS-40	Indonesia	−24.49 ± 0.06	−27.33 ± 0.06	***2***.***84***^***b***^	***16***.***13***^***c***^
AS-41	Iran*	−***17***.***47***^***a***^ ± ***0***.***22***	−22.30 ± 0.20	***4***.***83***^***b***^	***38***.***30***^***c***^
AS-42	Iran	−***13***.***35***^***a***^ ± ***0***.***07***	−23.07 ± 0.12	***9***.***71***^***b***^	***72***.***66***^***c***^
AS-43	Iran*	−***17***.***42***^***a***^ ± ***0***.***04***	−22.94 ± 0.13	***5***.***52***^***b***^	***41***.***72***^***c***^
AS-44	Iran	−***19***.***45***^***a***^ ± ***0***.***17***	−23.07 ± 0.14	***3***.***63***^***b***^	***27***.***13***^***c***^
AS-45	South Korea*	−***20***.***94***^***a***^ ± ***0***.***16***	−27.53 ± 0.20	***6***.***58***^***b***^	***36***.***93***^***c***^
AS-46	China	−***15***.***50***^***a***^ ± ***0***.***02***	—	—	—
EU-47	Greece*	−24.31 ± 0.10	−25.67 ± 0.14	***1***.***36***^***b***^	***8***.***50***^***c***^
EU-48	Hungry	−24.77 ± 0.06	−25.97 ± 0.16	***1***.***19***^***b***^	***7***.***32***^***c***^
EU-49	Macedonia*	−***17***.***46***^***a***^ ± ***0***.***08***	−22.49 ± 0.03	***5***.***03***^***b***^	***39***.***30***^***c***^
EU-50	Macedonia*	−***17***.***68***^***a***^ ± ***0***.***10***	−23.13 ± 0.14	***5***.***44***^***b***^	***40***.***55***^***c***^
EU-51	Romania*	−24.84 ± 0.004	−25.92 ± 0.12	***1***.***08***^***b***^	6.64
EU-52	Serbia*	−***17***.***37***^***a***^ ± ***0***.***04***	—	—	—
OA-53	New Zealand*	−25.13 ± 0.09	−23.66 ± 0.06	−1.47	−***10***.***55***^***d***^
OA-54	New Zealand*	−25.58 ± 0.20	−26.78 ± 0.06	***1***.***20***^***b***^	***7***.***04***^***c***^

Values in bold indicate honey samples that did not meet the specific criteria.

Data are expressed as Mean ± 1SD with triplicates, and “—” means no extractable protein.

*International honeys obtained from local food markets and commercial supermarkets in Australia. Manuka honey (OA-53) had the MGO (methylglyoxal) value of 30+ (mg/kg) on its label; manuka honey OA-54 had no MGO or NPA (non-peroxide activity) information on its label.

^a^Detection criterion for δ^13^C_honey_ <−23.5‰ according to the AOAC Official Method 978.17^10^.

^b^Detection criterion for δ^13^C_h-p_ ≤1‰ according to Padovan *et al*.^8^, White and Winters^15^, Simsek *et al*.^17^, Tosun^18^, Guler *et al*.^19^, Elflein and Raezke^20^.

^c^Detection criterion for C-4 sugar ≤7% according to the AOAC Official Method 998.12^11^.

^d^Detection criterion for C-4 sugar where >−7% according to Dong *et al*.^12^.

According to the AOAC 991.41^16^ along with corresponding studies^8,15,17–20^, the value of δ^13^C of the honey and its protein should differ by no more than 1‰ (δ^13^C_h-p_ ≤ 1‰), which is equivalent to <7% added corn or cane sugar. In this study, 19 honey samples (20% of total commercial samples analysed) had δ^13^C values in their honey and protein that were >1‰ (1.07–9.71‰) (Table 1). Two samples (AS-46 and EU-52) had no extractable protein (Table 1) but their honey carbon isotopic ratios −15.50 and −17.37‰ where >−23.5‰ indicated they had been adulterated with C-4 sugars, such as those from corn or sugar cane^10^.

In addition to the above standards to detect authentic honey, a recent study by Dong *et al*.^12^ indicated that honey with C-4 sugar content <−7% ought to be also classified as being adulterated. Two Australian samples marginally exceeded this criterion (M-AUS-25, −7.66%; M-AUS-26, −7.02%) along with two overseas samples (AS-36, −11.04%; OA-53, −10.55%), indicating that they had been potentially adulterated (Table 1).

**Figure 1:**
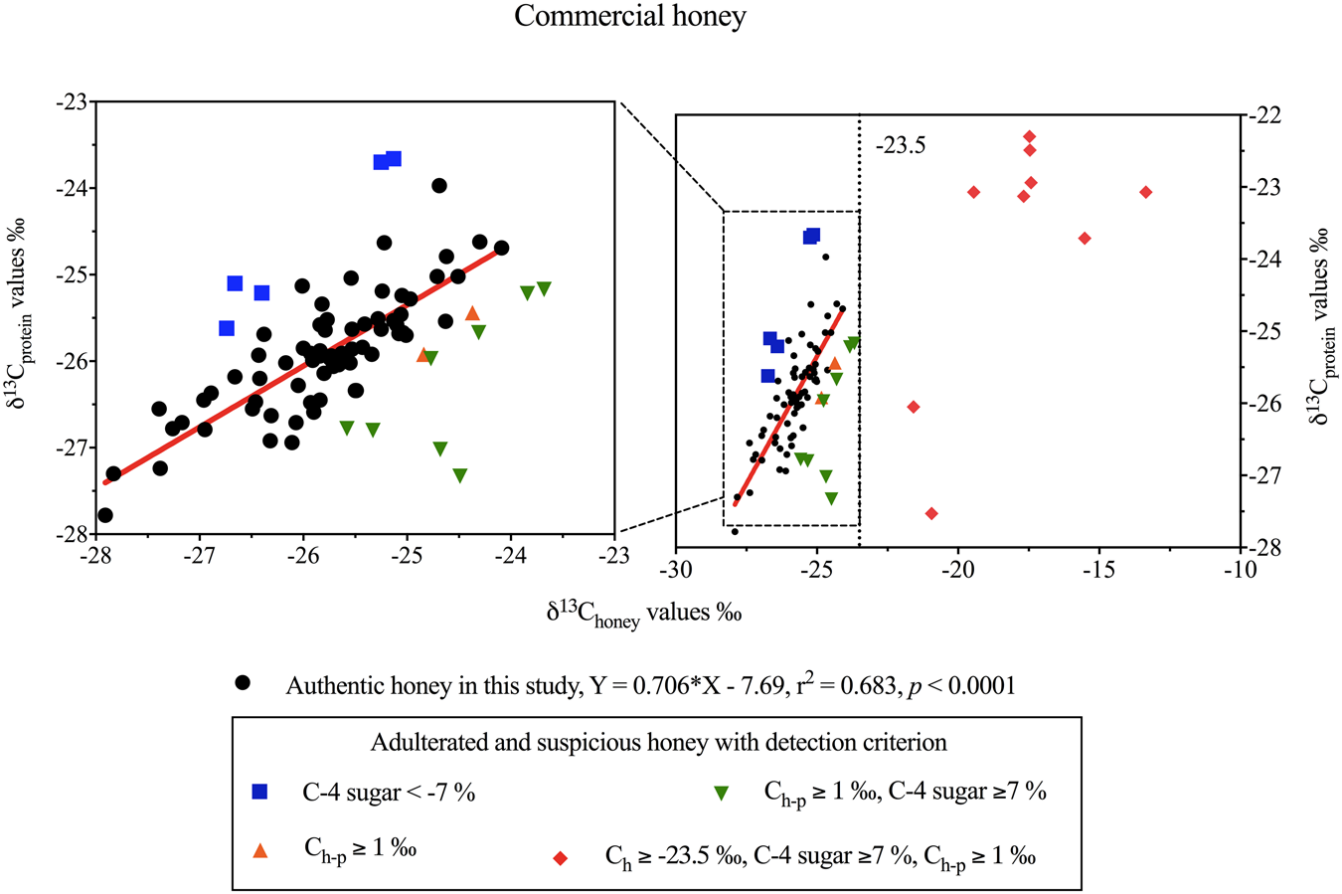
Bivariate plot of δ^13^C_honey_ and δ^13^C_protein_ values of commercial honey from Australia (mainland and Tasmania) (n = 38), overseas (n = 54) and unknown origin (n = 3). Circles represent authentic honey (Supplementary Table S1); triangles, squares and diamonds represent adulterated honey (Table 1).

The adulterated honeys (n = 26, 27% of the total (n = 95) commercial honey samples) had δ^13^C values ranging from −26.74 to −13.35‰ in honey and −27.53 to −22.30‰ in protein. By contrast, the range of δ^13^C values in pure honeys (n = 69) was much narrower from −27.91 to −24.09‰ in honey and −27.78 to −23.97‰ in protein, resulting in a regression analysis of δ^13^C_protein_ = 0.706 × δ^13^C_honey_ − 7.69 (R^2^ = 0.683, *p* < 0.0001) (Fig. 1).

### Trace element analysis

**Figure 2:**
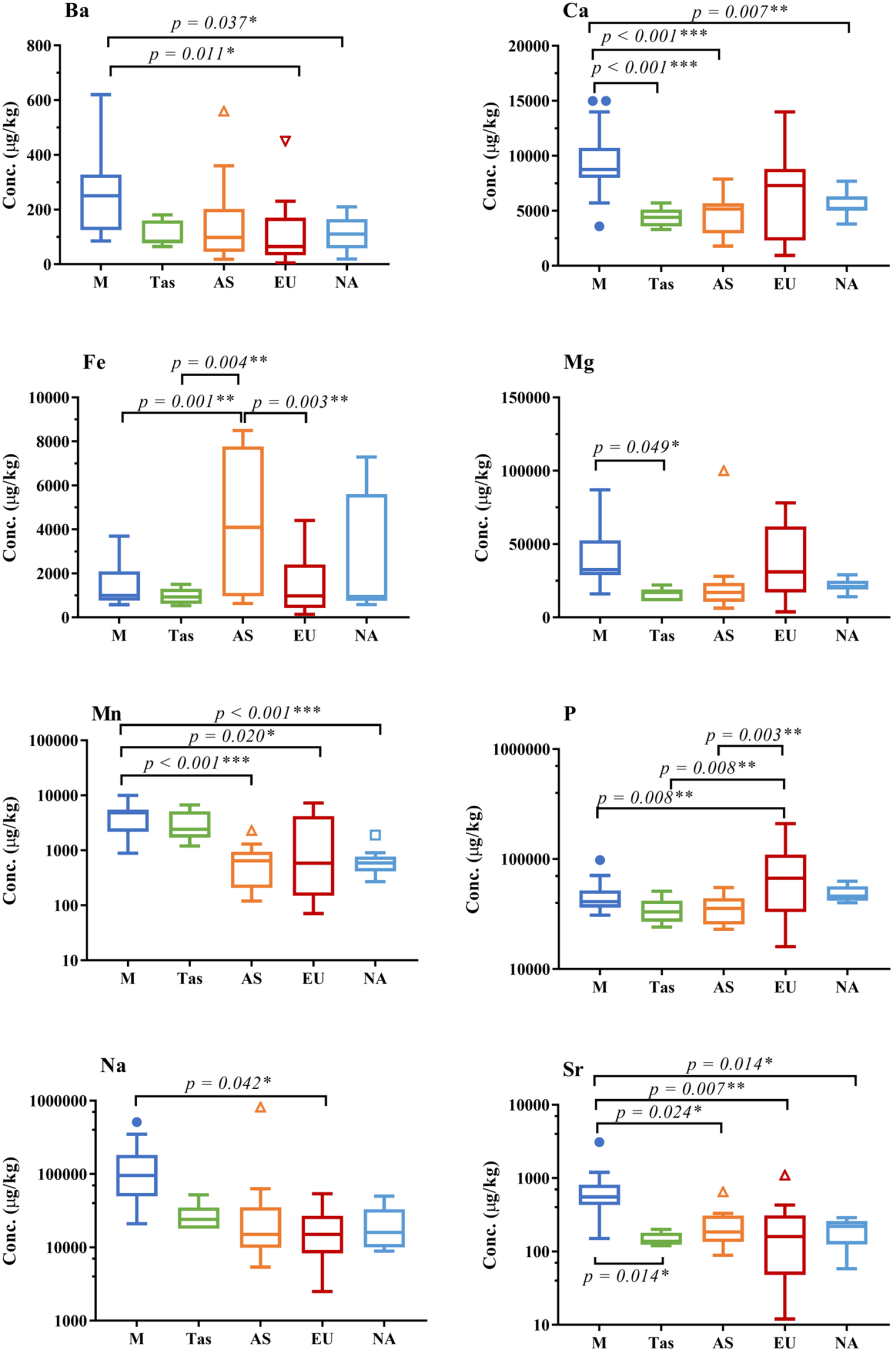
Boxplot with Tukey whiskers showing trace element concentrations (µg/kg) of Ba, Ca, Fe, Mg, Mn, P, Na and Sr for authentic commercial honey samples of known geographic origin (n = 65; with the three samples of an unknown origin and the single African honey sample excluded) collected from mainland Australia (n = 24), Tasmania (n = 7), Asia (n = 10), Europe (n = 15) and North America (n = 9). Significant differences were determined using a One-way ANOVA with Tukey’s multiple comparison at ***p = 0.001, **p = 0.01 and *p = 0.05 levels.

The 69 commercial honeys that passed the C-4 sugar criteria were further analysed for their trace element concentrations. The 69 authentic honeys were from mainland Australia (n = 24), Tasmania (n = 7), Asia (n = 10), Europe (n = 15), North America (n = 9) and Africa (n = 1), with three samples having no clear geographic information on their label. One-way analysis of variance (ANOVA) followed by a Tukey’s multiple comparison was used to determine the difference between each pair of geographic categories (three samples of an unknown origin and one African honey were excluded) with significance being identified when *p* < α = 0.05 (Fig. 2). The full dataset of trace element concentrations and *p* values between different geographic groups is provided in Supplementary Tables S2 and S3, respectively.

There were significant differences between honey samples from mainland Australia and Tasmania in the trace elements Ca (*p* < 0.001), Mg (*p* = 0.049) and Sr (*p* = 0.014). Authentic commercial honey samples from mainland Australia and overseas (Asia, Europe and North America) had significant differences (*p* = <0.001–0.042) in the trace elements Ba, Ca, Fe, Mn, P, Na and Sr. However, Tasmania honey had significantly different concentrations in Fe (*p* = 0.004) and P (*p* = 0.008) compared to international honey from Asia and Europe only. Asian and European honey also showed significant differences (*p* = 0.003) in the trace elements Fe and P (Fig. 2).

### PCA and CDA analysis

**Figure 3:**
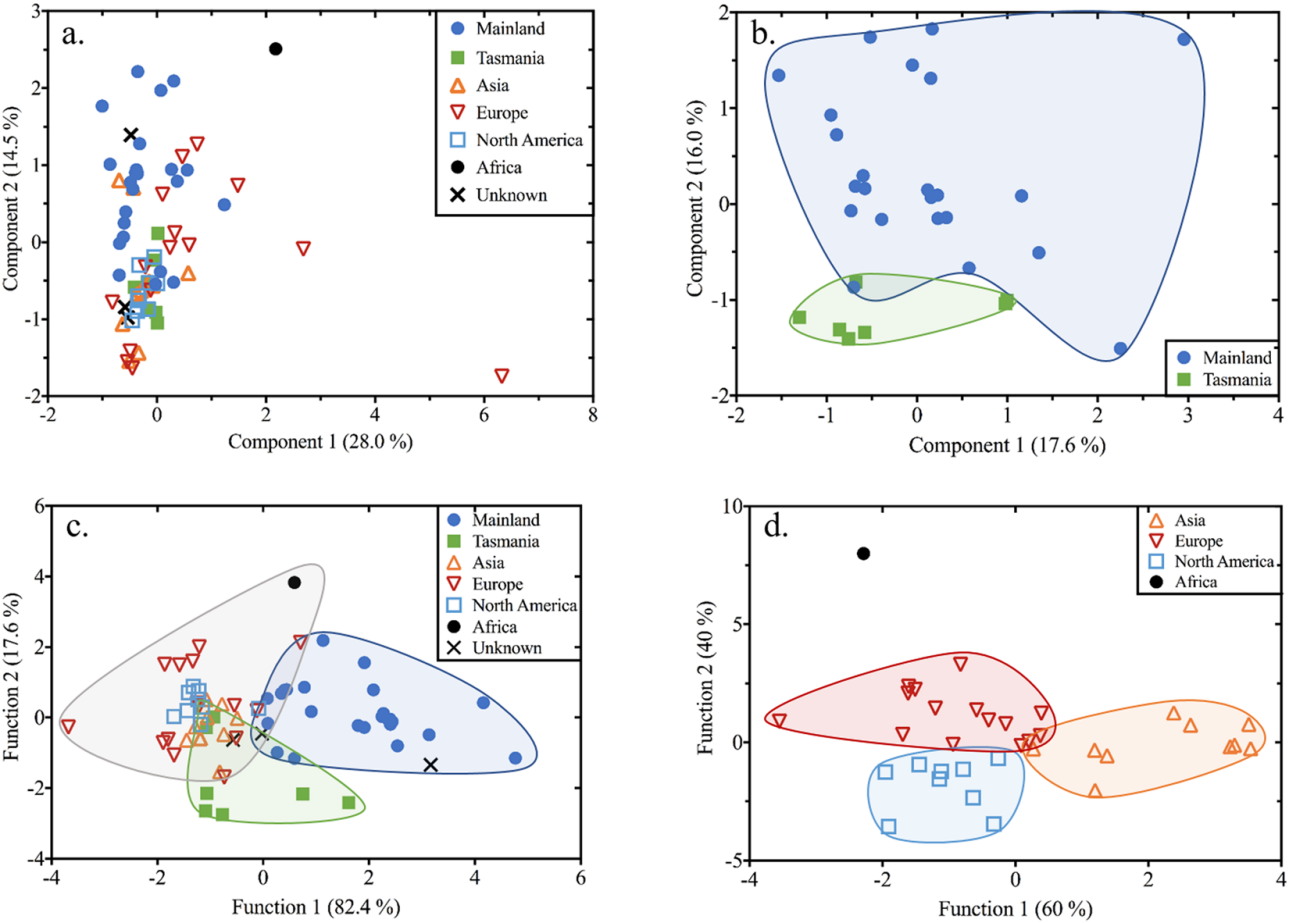
Score plots of PCA (**a**,**b**) and CDA (**c**,**d**) analysis used to distinguish all authentic honey samples (n = 69) from mainland Australia (n = 24), Tasmania (n = 7), Asia (n = 10), Europe (n = 15), North America (n = 9), Africa (n = 1) and unknown geographic origin (n = 3). (**a**) Important parameters are Cu, Ni, P and Rb in Component 1, and Ba, Ca, Mg and Mn in Component 2. (**b**) Important parameters are δ^13^C_honey_, δ^13^C_protein_ and Ba in Component 1, and Ca, Mg and Na in Component 2. (**c**) Honey from mainland Australia (blue shaded area), Tasmania (green shaded area) and overseas (grey shaded area) were used as the input groups to train the CDA model. CDA was then used to predict the geographic origin of the three honeys produced in an unknown geographic location (Supplementary Table S4). The important parameters are Mn and Sr in Function 1, and Ca, P and K in Function 2. (**d**) Honey from Europe (in red shaded area), Asia (yellow shaded area) and North America (light blue shaded area) were used as the input groups. The important parameters are Ba, Ca, Fe, Na and Sn in Function 1, and δ^13^C_honey_, δ^13^C_protein_, Al, B, Cu, K, Mg, Mn, Ni, P, Rb, Sr and Zn in Function 2. Loading values for the PCA and CDA results are in Supplementary Table S5 and S6, respectively.

Trace elements Ba, Ca, Fe, Mg, Mn, P, Na and Sr showed statistical differences between honey samples according to their geographic origin (Fig. 2). However, a combination of any two of these trace elements alone could not be used to visually depict geographic origin (Supplementary Fig. S1). Therefore, multivariate methods of principal component analysis (PCA) (Fig. 3a,b) and canonical discriminant analysis (CDA) (Fig. 3c,d) were applied to reduce the number of variables needed to describe the variation between individual samples, resulting in group differences being visible with a low-dimensional view for geographic classification.

PCA generates principal components that are linear combinations of the original variables. The PCA results for all seven geographic groups (mainland Australia, Tasmania, Asia, Europe, North America, Africa and unknown origin) showed that the first six components accounted for 77.2% of the total variability, with no visual clustering according to honey’s geographic origin in the first two components (Fig. 3a). Interestingly, PCA results for honeys from mainland Australia and Tasmania showed the first six components accounted for 81.5% of the total variability, with clustering evident in the first two components (Fig. 3b).

In contrast to the PCA, CDA produced clear groupings for Australia and overseas (Fig. 3c). Also, international honeys were further separated according to their respective continental origins of Asia, Europe, North America and Africa (Fig. 3d). The most important parameters to separate Australian honey (mainland and Tasmania) from overseas honey were Ca, Mn, P, K and Sr (Fig. 3c). Classification results from CDA showed that 84.8% of the original grouped cases were correctly classified according to their different geographic origin (Supplementary Table S4). Cross validation analysis returned a 75.8% correction rate, revealing that CDA is a reliable and suitable model for predicting the geographic origin of honey (Supplementary Table S4). Also, the classification results indicated that one of the unknown origin honey samples was from mainland Australia with the other two being grouped with overseas samples (Fig. 3c and Supplementary Table S4).

### Model analysis

The PCA and CDA displayed a clear visual clustering for honeys according to their different geographic origin covering both regions (Fig. 3b,c) and continents (Fig. 3d). In order to identify a potential approach for distinguishing honey from different regions and continents, a C5.0 classification model with 92.3% overall accuracy was applied to honey carbon isotopic values and trace element concentrations. This model was used to test its predictive accuracy to correctly group honeys according to its geographic origin. Sixty-five authentic commercial honey samples with clear geographic information were used for model analysis (the single African sample was excluded). Training (90% sample size, n = 60) and testing sets (10% sample size, n = 5) were used to build and evaluate the model. The training set is used to train the candidate algorithms, and the testing set is used to measure the predictive ability of the model. Results from both sets showed 100% classification rates in grouping honey from different geographic origins according to the C5.0 model.

**Figure 4:**
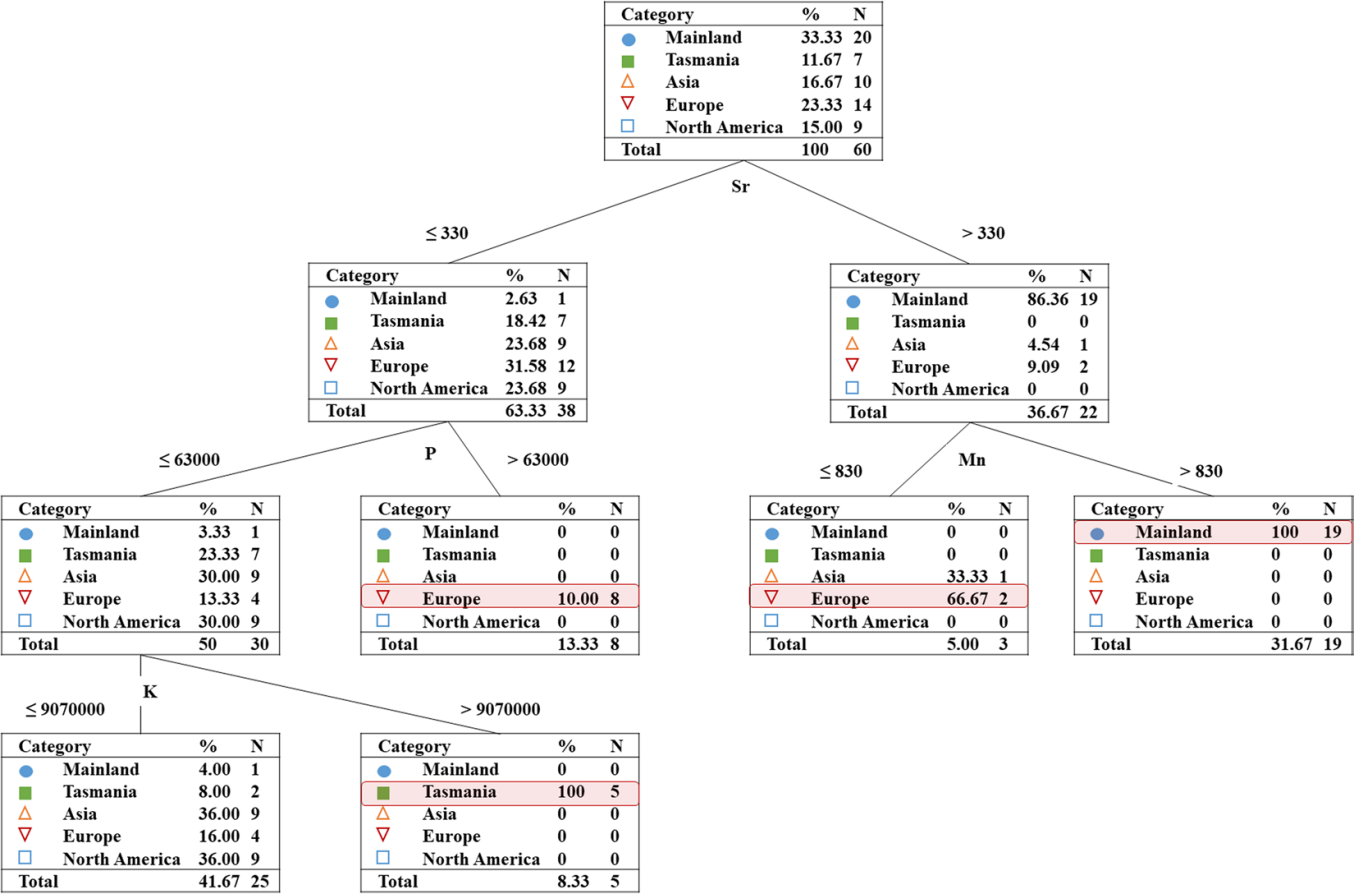
Decision tree outcomes (partial results) with four end nodes to separate honey according to concentrations (µg/kg) of Sr, P, Mn and K. Full results are provided in Supplementary Fig. S2.

Trace element profiles in honey revealed predictor importance in the C5.0 classification model (Fig. 4 and Supplementary Fig. S2). The C5.0 model demonstrated that trace elements Sr, P, Mn and K can be used to differentiate 95% (19 of 20) samples from the Australian mainland, 71% (5 of 7) samples of Tasmanian, and 71% (10 of 14) samples of European honey (Fig. 4) in four end nodes.

## Discussion

Honey is ranked third in the list of globally adulterated products^43^. The common fraudulent practice of adulterating commercial honey is confirmed by this study of global honeys (Supplementary Fig. S3). We found that 52% of Asian honey samples tested were adulterated (11 of 21 samples) (Table 1); of which three were from China (3/7), one from South Korea (1/1), one from India (1/2), two from Indonesia (2/2) and four from Iran (4/4). Six honey samples from Europe, from a total of 21 tested, contained added sugar. These honeys originated from Macedonia (2/3), Romania (1/2), Serbia (1/1), Greece (1/5) and Hungary (1/3). Australia has a lower adulteration rate (18.4% in total), with honey from its mainland having an adulteration rate of 17.2% (5/29) compared to 22.2% of samples from Tasmania (2/9). Both New Zealand manuka honey samples tested (2/2) were adulterated.

Manuka honey is prone to failing the C-4 sugar test carried out using the AOAC 998.12^11^, as it can present “false-positive” results for sugar adulteration as a result of insoluble material (such as pollen or dust) being retained in honey or due to the removal of pollen by filtration and centrifuging, affecting its δ^13^C protein value^44–47^. Manuka sample OA-53 returned a value of −10.55% for C-4 sugar (<−7% cf. Dong *et al*.^12^) and OA-54 had a 7.04% C-4 sugar value (>7% cf. AOAC 998.12^11^) and 1.20‰ in δ^13^C_h-p_ (>1‰)^8,15,17–20^, classifying them as adulterated. Moreover, the manuka samples had δ^13^C_honey_ values of −25.13‰ and −25.58‰ respectively, which is inconsistent with Rogers *et al*.^45^ who suggested that δ^13^C values in authentic manuka honey should not be less than −24.7‰. Therefore, the multiple criteria used here to assess honey authenticity indicates that both the manuka honey samples analysed were non-authentic products. Rogers *et al*.^45^ also analysed manuka honey samples and found that 15% (113 of 757) were adulterated.

Stable carbon isotopic ratio analysis has gained increasing importance in the determination of the geographic origin of honey^21^. The δ^13^C values of honey and protein are strongly influenced by climatic conditions^48^ and agricultural practices^19^. Nine honey samples were collected from the island state of Tasmania, 240 km south of the Australian continental mainland. The different geographic environment of mainland Australia and Tasmania appears to be reflected in the carbon isotope ratios of their respective honey and honey protein values. The mean values of δ^13^C_honey_ in authentic Australian honey samples were Tasmania (−25.57‰) > mainland (−25.85‰) > raw honey (−26.15‰) (Supplementary Table S1). These three groups of Australian honey that ranged from −27.91‰ to −24.71‰, correspond to the range determined by the Australian government^49^ who established authentic honeys had δ^13^C values from −27.47‰ to −24.28‰. Tasmanian honey (−25.68‰) also has higher mean δ^13^C values for protein than Australian mainland honey (−26.08‰) and raw honey (−25.79‰) (Supplementary Table S1). The δ^13^C_protein_ (−27.78‰ to −25.02‰) for all authentic Australian honeys (comprising raw, and commercial mainland Australia and Tasmania samples) also corresponds with authentic honeys identified in the Australian government investigation (−27.20‰ to −23.22‰)^49^. Hence, the differences in carbon isotopes are related not only to the source locations but also to whether the honey is raw or has been commercially processed.

Trace element analysis can also be used to discriminate the geographic origin of food^50^. However, two or three individual trace elements are typically ineffective for differentiating foods according to their geographic origin (Supplementary Fig. S1)^51^. Hence, multivariate statistical methods can provide better discrimination between geographic regions^52^. PCA and CDA are both commonly used statistical methods to reduce the dimensions of variability for clustering. CDA is a supervised learning method that can classify unknown samples (Supplementary Table S4). In this study, CDA resulted in more effective clustering than PCA as it was able to separate authentic commercial honey samples according to their geographic origin, similar to Kropf *et al*.^37^, Efenberger-Szmechtyk *et al*.^53^, and Zakaria *et al*.^54^ in their analysis of the geographic classification of honey.

Advanced statistical analyses, such as decision trees, have been used previously to verify the most relevant trace elements for classifying the geographic origin of food^50,51,55^. In this study, we applied the C5.0 classification model to separate honey at regional and continental scales: that is, samples originating from mainland Australia, Tasmania, and also Asia, Europe and North America. The C5.0 classification model is consistent with the CDA statistical analysis in that they both show elemental concentrations of Mn and Sr in honey can be used to distinguish honeys from mainland Australia from those sourced from Tasmania and overseas (95% of samples and 82.4% of the total variation, respectively) (Fig. 3c and 4). The trace elements Mn and Sr together with K and P can be used to separate 71% of Tasmanian honey (5 in 7) from the other samples with a specific geographic identity. This outcome was also reflected in the CDA analysis where the same four elements were included in Function 1 (Mn and Sr) and Function 2 (K and P) (Fig. 3c). Further, the same four elements were also included in the second function for classifying different continental honey (Fig. 3d). This is consistent with the C5.0 classification model that showed 71% (10 in 14) of European honeys were differentiated from samples from Asia, North America, Tasmania and mainland Australia (Fig. 4). Other studies have also shown that the trace elements K^36,56^, Mn^56,57^, P^58^ and Sr^36,59^ were critical for the classification of honey according to their geographic origin.

This study investigated the authenticity of global commercial honeys, covering 19 countries from five continents. The issue of authentication of honey cannot be ignored in the international honey market, not only in Asian countries but also European^60^ and Oceanic countries. Analyses of δ^13^C values in honey and its protein can be an effective tool for the authentication of honey where it has been subject to adulteration with C-4 sugars. Further, analysis of trace elements in honey for fingerprinting its geographic origin shows significant potential to assist in product authentication. Thus, this study shows that a combination of δ^13^C and statistical analysis of trace element concentrations can be used to expose the common fraudulent practice of adding C-4 sugars along with the mislabelling of a honey’s geographic origin.

## Limitations

This study contains a number of potential limitations. Firstly, the study is comprised of a relative small sample size (n = 100). The study was not intended to be a systematic survey of global honeys, but rather a snapshot of randomly sampled honeys to ascertain the prevalence of adulteration. Indeed, the results presented here are consistent with other assessments of honey adulteration rates^60^. While the analysis of honey’s trace element concentrations showed clear potential to discriminate between samples from different geographic locations, a larger sample size would be required to further confirm this study’s preliminary findings. However, to fully characterise the variance of trace elements across a region may require prohibitively expensive large data sets. An alternative lower cost approach could involve establishing if trace element concentrations of an unknown product are consistent with genuine reference products and are different to those sourced from potential risk locations.

While the study showed that some 73% of commercial honeys analysed were classified as pure according to AOAC criteria^10,11,16^ and previous studies^8,12,13,15,17–20^ it is entirely possible this overestimated the actual number of pure honeys. This is because honey is sometimes adulterated using C-3 sugars, such as those from sugar beet^7^. It is unfortunate that the methods used here cannot detect this form of adulteration^61^. Consequently, detection of adulteration of this nature remains a challenge^7^.

In addition to C-3 sugars, other adulterants in honey are difficult to detect due to the development of new and more sophisticated practices and lack of officially accepted analytical techniques. The use of EA-IRMS (Elemental Analysis - Isotope Ratio Mass Spectrometry) is the only official detection method for addition of C-4 sugar in honey^42^. This study also relied on a number of AOAC criteria^10,11,16^ to determine if honey samples were pure. As shown in Table 1, 65% (17 of 26) of samples classified as adulterated did not meet the δ^13^C_h-p_ and the C-4 sugar criteria; 9 samples also failed to meet the relevant criterion for δ^13^C_honey_ < −23.5‰^10^. While the C-4 sugar criterion is considered robust and has broad application, the AOAC 998.12^11^ method does identify that a small percentage of genuine pure honeys fall outside of the accepted criterion for non-adulterated honey (≤7%).

## Method

### Sample collection

Raw and unprocessed honey samples (n = 5) were collected for this study from beehives across Sydney, New South Wales (*Apis mellifera*, n = 3) and Calliope, Queensland (*Tetragonula hockingsi*, n = 2), Australia. Commercial honeys were obtained from local food markets and commercial supermarkets. Of the twenty-nine Australian mainland commercial honeys (M) labelling information indicated two were from NSW, six from Queensland, seven from Victoria, six from Western Australia, four from South Australia, with four of the Australian honeys not providing state of origin information on their labels. A further nine Australian Tasmanian honey samples (TAS) were collected for analysis.

Fifty-four commercial honey samples were acquired overseas from five continents; Africa (n = 1 from Kenya), Asia (AS, n = 21), Europe (EU, n = 21), North America (NA, n = 9) and Oceania (OA, n = 2 from New Zealand). Asian countries investigated in this study include China (n = 7), India (n = 2), Indonesia (n = 2), Iran (n = 4), Japan (n = 3), Saudi Arabia (n = 2) and South Korea (n = 1). European commercial honeys were collected from England (n = 3), Greece (n = 5), Hungary (n = 3), Italy (n = 4), Macedonia (n = 3), Romania (n = 2) and Serbia (n = 1). Honey from USA (n = 7) and Canada (n = 2) were included as North American samples. Nineteen of the overseas honey samples were purchased in Australian supermarkets (marked with an asterisk in Supplementary Table S1 and Table 1) with the remainder being acquired from overseas. Three commercial honey samples with an unclear origin of production (U) were purchased in Australia. Two had their origin listed as “made in Australia from local and imported ingredients”, with the other having no geographic identification. All samples (raw n = 5, and commercial honey samples n = 95) were analysed at the National Measurement Institute (NMI), North Ryde, Sydney.

### Sample preparation and analysis

#### Trace element analysis

One gram of honey was digested with 3 mL HNO_3_ before heating at 100 °C for 2 h. Each digested sample was topped up to 40 mL with Milli-Q (18.2 MΩ.cm) deionised water. Samples were diluted two times prior to analysis for their trace element concentrations on an Inductively Coupled Plasma Mass Spectrometer (Agilent 7900 equipped with an Integratated Sample Introduction System). Each sample batch (n = 20) contained a laboratory reagent blank and duplicate, blank spike, blank matrix, duplicate sample and matrix spikes. Sixty-four trace elements were measured in the samples: Ag, Al, As, Au, B, Ba, Be, Bi, Ca, Cd, Ce, Cs, Cr, Co, Cu, Dy, Er, Eu, Fe, Ga, Gd, Ge, Hg, Hf, Ho, Rb, K, La, Li, Lu, Mg, Mn, Mo, Na, Nb, Nd, Ni, Os, P, Pb, Pd, Pt, Pr, Re, Ru, Se, Sb, Sr, Sm, Sn, Ta, Tb, Te, Th, Tl, Tm, Ti, U, V, W, Y, Yb, Zn and Zr. Forty eight of these trace elements were below the NMI’s Limit of Reporting (LOR) of 10 µg/kg in 70–100% of samples. As a result, the concentrations of 15 trace elements (Ba, B, Ca, Cu, Fe, Mg, Mn, Ni, P, K, Rb, Na, Sr, Sn and Zn) that were above the LOR in 71–100% of samples were used in the statistical analyses. Although only 46% of samples had Al at concentrations >LOR, its exclusion had potential to cause erroneous statistical outcomes because of its median concentration (760 µg/kg) and large range (23–21000 µg/kg). In all cases, sample procedural blanks were below the NMI’s LOR. The combined mean RSD (relative standard deviation) for the individual trace elements in honey was 2.4%. Matrix spike recovery rates for all trace elements in honey was 75–110%. Analytical uncertainties for all trace elements was 14–24%.

#### Carbon isotopic ratio analysis

Proteins from honey were isolated according to the AOAC Official Method 998.12^11^. Honey samples (10–12 g) were weighed into a centrifuge tube (50 mL) and mixed with 4 mL Milli-Q water, followed by the addition of 2 mL of 0.335 mol/L H_2_SO_4_ and 2 mL of Na_2_WO_4_ 10% (w/v). This mixture was homogenized and heated to 80 °C until flocculation of proteins were visible and a clear supernatant observed. In cases where flocculation did not occur, H_2_SO_4_ (2 mL, 0.335 M) was added to the solution. Sample tubes were filled to 50 mL with Milli-Q water, mixed thoroughly and centrifuged at 1500 × g for 5 min. The supernatant was subsequently discarded. This procedure was repeated for a minimum of five times, until the supernatant was clear. The precipitated protein was dried in an oven (75 °C) for a minimum of 3 h until dry.

Bulk honey (0.55–0.60 mg) and extracted protein (0.48–0.57 mg) were weighed into tin capsules (3.3 mm × 5 mm; IVA Analysentechnik, Meerbusch, Germany) and introduced into a Flash Elemental Analyzer coupled to an Isotope Ratio Mass Spectrometer (EA-IRMS, Thermo Finnigan Delta V Plus) via a ConFlo IV interface. Data was acquired using ISODAT 3.0 (Version 2.84) (ThermoScientific, Bremen, Germany). High purity oxygen (>99.5%), ultra-high purity helium (>99.99%), high purity carbon dioxide (>99.5%) were obtained from BOC gases (Sydney, Australia) and zero enrichments were performed for CO_2_ reference gas prior to sequence acquisitions. The δ-values of nine 20 s gas pulses were measured, and the standard deviation determined to be less than 0.2‰.

A typical sample sequence for δ^13^C analysis was initiated with the thermal combustion of two blank tin cups to ensure the system was void of contamination. Each honey and protein sample were analysed in triplicate alongside standards USGS 24 graphite (δ^13^C −16.05‰) and NBS 22 oil (δ^13^C −30.03‰), which returned mean RSDs of 0.36% and 0.33%, respectively. Three reference materials were employed as quality control samples in this study: CCQM-K140 (δ^13^C −24.09‰; analysed 6 times) returning a RSD of 0.27% and measurement uncertainties ranging between 0.08% and 0.28%[62]; USGS 40 L-glutamic acid (δ^13^C −26.39‰) and IAEA-CH-3 cellulose (δ^13^C −24.72‰) were analysed in quadruplicate and had a mean RSD of 0.27% and 0.35%, respectively.

### Data processing

The isotope ratios of ^13^C/^12^C in honey and protein are expressed as δ^13^C in units of ‰. Corrected values were obtained according to equation (2):

2 $${\delta }^{13}{\rm{C}}({\rm{\textperthousand }})=(\frac{{R}_{means(sample)}-{R}_{means(Std1)}}{{R}_{means(Std2)}-{R}_{means(Std1)}})\times ({R}_{true(Std2)}-{R}_{true(Std1)})+{R}_{true(Std1)}$$

where R = ^13^C/^12^C, R_means (sample)_ is the measured ^13^C/^12^C ratios of the honey or protein samples, R_means (Std 1)_ and R_means (Std 2)_ are the measured ^13^C/^12^C ratios of the standards USGS 24 graphite and NBS 22 oil, respectively; R_true (Std 1)_ and R_true (Std 2)_ are the true ^13^C/^12^C ratios for USGS 24 graphite and NBS 22 oil, which are −16.05‰ and −30.03‰, respectively.

Data were analysed using GraphPad Prism 7 for boxplot presentation with Tukey whiskers and a one-way ANOVA test. Multivariate approaches were used to investigate the feasibility of characterising the geographic origin of authentic commercial honey samples based on the measured variables of δ^13^C_honey_ and δ^13^C_protein_ together with trace elements of Al, Ba, B, Ca, Cu, Fe, Mg, Mn, Ni, P, K, Rb, Na, Sr, Sn and Zn. Principal component analysis and canonical discriminant analysis were carried out using IBM SPSS v22.0, and classification analyses using C5.0 model were implemented via IBM SPSS Modeler v18.0. Concentrations of trace elements with <LOR (10 µg/kg) were considered as 5 µg/kg for statistical analyses.

## Electronic supplementary material

Supplementary Information
