# Supplementary material for: Authenticity and geographic origin of global honeys determined using carbon isotope ratios and trace elements
Source: Sci Rep. 2018 Oct 2;8:14639. doi: 10.1038/s41598-018-32764-w (PMC6168535; doi:10.1038/s41598-018-32764-w)
Supplement: Supplementary file 1 — Supplementary Information [file 41598_2018_32764_MOESM1_ESM.docx]

Authenticity and geographic origin of global honeys determined using carbon isotope ratios and trace elements

Xiaoteng Zhou^1,*^, Mark Patrick Taylor^1,2,^**^*^**, Helen Salouros**^3^**, Shiva Prasad^4^

^1^Department of Environmental Sciences, Faculty of Science and Engineering, Macquarie University, North Ryde, Sydney, New South Wales, 2109, Australia

^2^Energy and Environmental Contaminants Research Centre, Macquarie University, North Ryde, Sydney, New South Wales, 2109, Australia

^3^Australian Forensic Drug Laboratory, National Measurement Institute, North Ryde, Sydney, New South Wales, 2113, Australia

^4^Analytical Service Branch, National Measurement Institute, North Ryde, Sydney, New South Wales, 2113, Australia

**Corresponding Authors**

*Email: [mark.taylor@mq.edu.au](mailto:mark.taylor@mq.edu.au) (M.P. Taylor), [xiaoteng.zhou@students.mq.edu.au](mailto:Xiaoteng.zhou@students.mq.edu.au) (X. Zhou)

**ORCID ID**

M.P. Taylor: <https://orcid.org/0000-0001-7598-9982>

X. Zhou: https://orcid.org/0000-0002-1900-6037

**Supplementary Table S1.** Data for δ^13^C_honey_ (‰), δ^13^C_protein_ (‰), δ^13^C_h-p_ (‰) and C-4 sugar (%) in raw honey samples (RAW, n = 5) and authentic commercial honey (n = 69) from mainland Australia (M-AUS, n = 24), Tasmania (TAS, n = 7), Africa (AF, n = 1), Asia (AS, n = 10), Europe (n = 15), North America (NA, n = 9) and honey of unknown origin (U, n = 3).

| **Sample No.** | **Country** | **δ^13^C_honey_ (‰)**  **Criterion:**  **<** −**23.5 ^a^** | **δ^13^C_protein_ (‰)** | **δ^13^C_h-p_ (‰)**  **Criterion:**  **≤ 1 ^b^** | **C-4 sugar (%)**  **Criterion:**  **≤ 7 ^c^ or > −7 ^d^** |
| --- | --- | --- | --- | --- | --- |
| RAW-1 | Australia | −25.65 ± 0.08 | −25.57 ± 0.10 | −0.08 | −0.50 |
| RAW-2 | Australia | −25.79 ± 0.04 | −25.57 ± 0.19 | −0.22 | −1.41 |
| RAW-3 | Australia | −26.90 ± 0.04 | −26.29 ± 0.14 | −0.61 | −3.71 |
| RAW-4 | Australia | −26.49 ± 0.03 | −26.06 ± 0.11 | −0.43 | −2.62 |
| RAW-5 | Australia | −25.90 ± 0.05 | −25.48 ± 0.02 | −0.42 | −2.69 |
| M-AUS-1 | Australia | −25.80 ± 0.05 | −26.14 ± 0.07 | 0.34 | 2.09 |
| M-AUS-2 | Australia | −25.66 ± 0.07 | −26.04 ± 0.12 | 0.38 | 2.35 |
| M-AUS-3 | Australia | −25.91 ± 0.07 | −25.99 ± 0.02 | 0.09 | 0.54 |
| M-AUS-4 | Australia | −25.74 ± 0.05 | −25.97 ± 0.003 | 0.22 | 1.38 |
| M-AUS-5 | Australia | −25.84 ± 0.01 | −26.45 ± 0.13 | 0.61 | 3.64 |
| M-AUS-6 | Australia | −25.41 ± 0.07 | −25.57 ± 0.03 | 0.16 | 0.98 |
| M-AUS-7 | Australia | −27.91 ± 0.05 | −27.78 ± 0.09 | −0.13 | −0.74 |
| M-AUS-8 | Australia | −26.07 ± 0.05 | −26.71 ± 0.18 | 0.64 | 3.76 |
| M-AUS-9 | Australia | −25.10 ± 0.02 | −25.58 ± 0.10 | 0.48 | 3.04 |
| M-AUS-10 | Australia | −25.90 ± 0.11 | −26.59 ± 0.17 | 0.70 | 4.12 |
| M-AUS-11 | Australia | −26.17 ± 0.09 | −26.02 ± 0.06 | −0.15 | −0.93 |
| M-AUS-12 | Australia | −25.71 ± 0.15 | −26.06 ± 0.13 | 0.35 | 2.16 |
| M-AUS-13 | Australia | −26.38 ± 0.10 | −25.69 ± 0.17 | −0.69 | −4.32 |
| M-AUS-14 | Australia | −27.83 ± 0.03 | −27.30 ± 0.14 | −0.53 | −3.03 |
| M-AUS-15 | Australia | −25.28 ± 0.07 | −25.51 ± 0.10 | 0.23 | 1.48 |
| M-AUS-16 | Australia | −26.49 ± 0.09 | −26.55 ± 0.09 | 0.06 | 0.34 |
| M-AUS-17 | Australia | −25.13 ± 0.10 | −25.53 ± 0.26 | 0.40 | 2.53 |
| M-AUS-18 | Australia | −25.05 ± 0.09 | −25.24 ± 0.06 | 0.19 | 1.20 |
| M-AUS-19 | Australia | −25.43 ± 0.07 | −25.84 ± 0.04 | 0.41 | 2.53 |
| M-AUS-20 | Australia | −25.06 ± 0.05 | −25.46 ± 0.07 | 0.40 | 2.56 |
| M-AUS-21 | Australia | −26.31 ± 0.06 | −26.63 ± 0.12 | 0.31 | 1.85 |
| M-AUS-22 | Australia | −25.84 ± 0.06 | −25.58 ± 0.02 | −0.26 | −1.61 |
| M-AUS-23 | Australia | −25.08 ± 0.08 | −25.68 ± 0.08 | 0.60 | 3.76 |
| M-AUS-24 | Australia | −25.34 ± 0.15 | −25.92 ± 0.08 | 0.58 | 3.55 |
| TAS-1 | Australia | −25.53 ± 0.01 | −25.63 ± 0.09 | 0.10 | 0.61 |
| TAS-2 | Australia | −25.82 ± 0.09 | −25.34 ± 0.14 | −0.48 | −3.09 |
| TAS-3 | Australia | −26.66 ± 0.06 | −26.18 ± 0.06 | −0.48 | −2.89 |
| TAS-4 | Australia | −25.77 ± 0.05 | −25.52 ± 0.06 | −0.25 | −1.59 |
| TAS-5 | Australia | −24.71 ± 0.12 | −25.02 ± 0.07 | 0.31 | 2.03 |
| TAS-6 | Australia | −25.49 ± 0.07 | −26.34 ± 0.22 | 0.85 | 5.11 |
| TAS-7 | Australia | −25.01 ± 0.04 | −25.70 ± 0.08 | 0.69 | 4.34 |
| **Sample No.** | **Country** | **δ^13^C_honey_ (‰)**  **Criterion:**  **<** −**23.5 ^a^** | **δ^13^C_protein_ (‰)** | **δ^13^C_h-p_ (‰)**  **Criterion:**  **≤ 1 ^b^** | **C-4 sugar (%)**  **Criterion:**  **≤ 7 ^c^ or >** −**7 ^d^** |
| AF-1 | Kenya | −24.69 ± 0.04 | −23.97 ± 0.04 | −0.72 | −5.05 |
| AS-1 | China | −25.24 ± 0.04 | −25.19 ± 0.21 | −0.05 | −0.32 |
| AS-2 | China | −25.04 ± 0.16 | −25.67 ± 0.17 | 0.63 | 3.95 |
| AS-3 | China | −24.30 ± 0.03 | −24.62 ± 0.05 | 0.31 | 2.11 |
| AS-4 | China | −24.62 ± 0.18 | −24.79 ± 0.09 | 0.17 | 1.12 |
| AS-5 | India | −26.89 ± 0.10 | −26.37 ± 0.10 | −0.53 | −3.17 |
| AS-6 | Japan | −27.38 ± 0.11 | −27.24 ± 0.04 | −0.14 | −0.81 |
| AS-7 | Japan | −27.26 ± 0.16 | −26.78 ± 0.14 | −0.48 | −2.81 |
| AS-7 | Japan | −25.54 ± 0.05 | −25.04 ± 0.07 | −0.49 | −3.21 |
| AS-9 | Saudi Arabia* | −25.79 ± 0.07 | −25.64 ± 0.23 | −0.15 | −0.96 |
| AS-10 | Saudi Arabia* | −24.63 ± 0.12 | −25.54 ± 0.05 | 0.92 | 5.78 |
| EU-1 | England | −26.96 ± 0.05 | −26.45 ± 0.06 | −0.51 | −3.04 |
| EU-2 | Europe | −26.43 ± 0.04 | −25.93 ± 0.07 | −0.50 | −3.10 |
| EU-3 | Europe | −26.32 ± 0.10 | −26.92 ± 0.04 | 0.60 | 3.48 |
| EU-4 | Greece* | −25.93 ± 0.06 | −26.48 ± 0.04 | 0.55 | 3.28 |
| EU-5 | Greece* | −25.84 ± 0.10 | −25.88 ± 0.05 | 0.04 | 0.25 |
| EU-6 | Greece* | −25.73 ± 0.01 | −25.94 ± 0.15 | 0.21 | 1.29 |
| EU-7 | Greece* | −25.93 ± 0.09 | −25.91 ± 0.07 | −0.02 | −0.13 |
| EU-8 | Hungary | −24.09 ± 0.10 | −24.69 ± 0.10 | 0.59 | 3.97 |
| EU-9 | Hungary | −26.01 ± 0.10 | −25.13 ± 0.04 | −0.88 | −5.70 |
| EU-10 | Italy* | −25.55 ± 0.08 | −26.02 ± 0.05 | 0.46 | 2.85 |
| EU-11 | Italy | −25.22 ± 0.03 | −24.63 ± 0.12 | −0.60 | −3.99 |
| EU-12 | Italy | −26.00 ± 0.03 | −25.85 ± 0.04 | −0.15 | −0.92 |
| EU-13 | Italy | −25.63 ± 0.08 | −25.91 ± 0.11 | 0.28 | 1.72 |
| EU-14 | Macedonia* | −24.51 ± 0.05 | −25.02 ± 0.16 | 0.51 | 3.34 |
| EU-15 | Romania* | −24.97 ± 0.003 | −25.28 ± 0.05 | 0.30 | 1.96 |
| NA-1 | Canada^e^ | −26.46 ± 0.14 | −26.47 ± 0.14 | 0.00 | 0.02 |
| NA-2 | USA | −25.84 ± 0.03 | −25.94 ± 0.10 | 0.10 | 0.61 |
| NA-3 | USA | −26.95 ± 0.06 | −26.79 ± 0.04 | −0.16 | −0.93 |
| NA-4 | USA | −26.42 ± 0.02 | −26.20 ± 0.03 | −0.22 | −1.31 |
| NA-5 | USA | −25.5 ± 0.03 | −26.34 ± 0.11 | 0.84 | 5.08 |
| NA-6 | USA | −26.11 ± 0.06 | −26.94 ± 0.02 | 0.82 | 4.78 |
| NA-7 | USA | −27.17 ± 0.09 | −26.71 ± 0.06 | −0.46 | −2.73 |
| NA-8 | USA | −26.05 ± 0.08 | −26.28 ± 0.08 | 0.23 | 1.40 |
| NA-9 | Canada | −25.70 ± 0.17 | −26.01 ± 0.08 | 0.32 | 1.95 |
| U-1 | Unknown* | −25.53 ± 0.09 | −25.86 ± 0.14 | 0.33 | 2.03 |
| U-2 | Unknown* | −25.25 ± 0.05 | −25.63 ± 0.01 | 0.37 | 2.34 |
| U-3 | Unknown* | −27.39 ± 0.04 | −26.55 ± 0.07 | −0.84 | −5.00 |

All Australian honey (mainland and Tasmania) was obtained from local food markets and commercial supermarkets.

*Nine authentic overseas honeys and three authentic honeys of an unknown origin were purchased in Australia.

^a^ Detection criterion for δ^13^C_honey_ <−23.5‰ according to the AOAC Official Method 978.17^1^;

^b^ Detection criterion for δ^13^C_h-p_ ≤1‰ according to Padovan et al.^2^, White and Winters^3^, Simsek et al.^4^, Tosun^5^, Guler et al.^6^, Elflein and Raezke^7^;

^c^ Detection criterion for C-4 sugar ≤7% according to the AOAC Official Method 998.12^8^;

^d^ Detection criterion for C-4 sugar where >−7% according to Dong et al.^9^;

^e^ Sample NA-1 was labelled as a product of USA-Canada but was purchased in Canada.

**Supplementary Table S2.** Concentrations of trace elements in authentic commercial honey samples (n = 69) from mainland Australia (n = 24), Tasmania (n = 7), Africa (n = 1), Asia (n = 10), Europe (n = 15), North America (n = 9) and honey with an unknown geographic origin (n = 3). Trace element concentrations are displayed to 3 significant figures. Arithmetic mean, error (1 standard deviation) and range are shown.

| **Trace elements** | | **Mainland Australia** | **Tasmania** | **Africa** | **Asia** | **Europe** | **North America** | **Unknown origin** |
| --- | --- | --- | --- | --- | --- | --- | --- | --- |
| Al (µg/kg) | Mean ± 1SD | 2120 ± 5280 | 1960 ± 1790 | 1400 | 89 ± 139 | 1860 ± 4720 | 227 ± 665 | < 10 |
|  | Range | < 10 – 21000 | < 10 – 4800 | -- | 5 – 440 | 5 – 18000 | 5 – 2000 | < 10 |
| Ba (µg/kg) | Mean ± 1SD | 257 ± 145 | 106 ± 45 | 700 | 157 ± 174 | 114 ± 118 | 110 ± 63.2 | 121 ± 42 |
|  | Range | 85 – 620 | 65 – 180 | -- | 18 – 560 | 5 – 450 | 19 – 210 | 94 – 170 |
| B (mg/kg) | Mean ± 1SD | 5.20 ± 1.75 | 4.84 ± 2.21 | 9.7 | 5.88 ± 3.15 | 4.97 ± 2.56 | 7.14 ± 0.87 | 4.13 ± 0.61 |
|  | Range | 2.50 – 10.0 | 3.30 – 9.20 | -- | 3 – 12 | 1.3 – 10 | 5.6 – 8.3 | 3.60 – 4.80 |
| Ca (mg/kg) | Mean ± 1SD | 9.38 ± 2.77 | 4.41 ± 0.89 | 12 | 4.77 ± 1.81 | 6.9 ± 4.1 | 5.58 ± 1.12 | 6.90 ± 4.46 |
|  | Range | 3.60 – 15.0 | 3.30 – 5.70 | -- | 1.8 – 7.9 | 0.95 – 14 | 3.8 – 7.7 | 3.70 – 12.0 |
| Cu (µg/kg) | Mean ± 1SD | 203 ± 168 | 126 ± 19 | 1.1 | 141 ± 144 | 314 ± 317 | 149 ± 47.9 | 73 ± 17 |
|  | Range | 65 – 770 | 100 – 160 | -- | 41 – 520 | 32 – 1300 | 88 – 230 | 54 – 86 |
| Fe (mg/kg) | Mean ± 1SD | 1.44 ± 0.94 | 0.93 ± 0.36 | 2.6 | 4.14 ± 3.09 | 1.4 ± 1.18 | 2.74 ± 2.78 | 2.63 ± 1.63 |
|  | Range | 0.58 – 3.70 | 0.54 – 1.50 | -- | 0.64 – 8.5 | 0.14 – 4.4 | 0.59 – 7.3 | 1.20 – 4.40 |
| Mg (mg/kg) | Mean ± 1SD | 40.1 ± 18.32 | 16.1 ± 4.10 | 75 | 24.7 ± 27.3 | 37 ± 25.1 | 22 ± 4.47 | 24.0 ± 16.5 |
|  | Range | 16.0 – 87.0 | 11.0 – 22.0 | -- | 6.3 – 100 | 3.8 – 78 | 14 – 29 | 14.0 – 43.0 |
| Mn (mg/kg) | Mean ± 1SD | 4.23 ± 2.29 | 3.14 ± 2.00 | 3.5 | 0.75 ± 0.65 | 2.13 ± 2.6 | 0.7 ± 0.49 | 3.23 ± 2.94 |
|  | Range | 0.89 – 10.0 | 1.20 – 6.70 | -- | 120 – 2300 | 0.07 – 7.3 | 0.27 – 1.9 | 1.20 – 6.60 |
| Ni (µg/kg) | Mean ± 1SD | 33 ± 42 | 25 ± 15 | 62 | 40.5 ± 54.1 | 132 ± 230 | 19.1 ± 8.85 | 10 ± 5 |
|  | Range | < 10 – 170 | < 10 – 53 | -- | 5 – 160 | 5 – 860 | 5 – 28 | < 10 – 15 |
| P (mg/kg) | Mean ± 1SD | 46.4 ± 15.7 | 34.3 ± 9.38 | 130 | 36 ± 10.7 | 76.8 ± 49.6 | 48.9 ± 8.51 | 25.0 ± 1.00 |
|  | Range | 31.0 – 98.0 | 24.0 – 51.0 | -- | 23 – 55 | 16 – 210 | 40 – 63 | 24.0 – 26.0 |
| K (mg/kg) | Mean ± 1SD | 8370 ± 3560 | 9620 ± 3518 | 7410 | 5020 ± 4660 | 10200 ± 8370 | 4340 ± 1750 | 6390 ± 6510 |
|  | Range | 2500 – 15800 | 4540 – 13000 | -- | 1.02 – 16.9 | 1620 – 26900 | 2100 – 7480 | 2370 – 13900 |
| Rb (mg/kg) | Mean ± 1SD | 1.84 ± 1.39 | 2.18 ± 0.78 | 4.9 | 0.89 ± 0.54 | 1.98 ± 2.41 | 1 ± 0.81 | 1.48 ± 1.49 |
|  | Range | 0.18 – 5.10 | 0.95 – 3.00 | -- | 0.14 – 1.8 | 0.16 – 7.4 | 0.22 – 3 | 0.56 – 3.20 |
| Na (mg/kg) | Mean ± 1SD | 137 ± 122 | 28.0 ± 12.4 | 5.8 | 99.7 ± 254 | 18.7 ± 14.3 | 21.3 ± 14.7 | 90.7 ± 60.2 |
|  | Range | 21.0 – 510 | 18.0 – 52.0 | -- | 5.4 – 820 | 2.5 – 54 | 8.9 – 50 | 52.0 – 160 |
| **Trace elements** | | **Mainland Australia** | **Tasmania** | **Africa** | **Asia** | **Europe** | **North America** | **Unknown origin** |
| Sr (µg/kg) | Mean ± 1SD | 683 ± 564 | 147 ± 30 | 890 | 242 ± 166 | 240 ± 268 | 195 ± 78.6 | 357 ± 272 |
|  | Range | 150 – 3100 | 120 – 200 | -- | 89 – 650 | 12 – 1100 | 58 – 290 | 180 – 670 |
| Sn (µg/kg) | Mean ± 1SD | 21 ± 14 | 25 ± 16 | 5 | 110 ± 313 | 17.5 ± 9.19 | 72.8 ± 128 | < 10 |
|  | Range | < 10 – 55 | < 10 – 58 | -- | 5 – 1000 | 5 – 35 | 4 – 380 | < 10 |
| Zn (mg/kg) | Mean ± 1SD | 1.46 ± 3.75 | 0.53 ± 0.31 | 1.1 | 1.25 ± 1.39 | 0.95 ± 0.62 | 1.52 ± 1.34 | 3.37 ± 5.14 |
|  | Range | 0.33 – 19.0 | 0.29 – 1.20 | -- | 0.37 – 4.9 | 0.18 – 2.4 | 0.32 – 4.3 | 0.23 – 9.30 |

**Supplementary Table S3**. *p* values of carbon isotopic ratios (‰) and 16 trace element concentrations in authentic commercial honey samples according to their different geographic origin (n = 65, with the three samples of unknown origin and the single African sample excluded). Samples were collected from mainland Australia (n = 24), Tasmania (n = 7), Asia (n = 10), Europe (n = 15) and North America (n = 9). Statistically significant differences (*p* = 0.001***, *p* = 0.01** and *p* = 0.05*) were determined using a One-way ANOVA with Tukey’s multiple comparison using the variables of carbon isotopic ratios (‰) of honey and its protein along with 16 trace element concentrations Al, Ba, B, Ca, Cu, Fe, Mg, Mn, Ni, P, K, Rb, Na, Sr, Sn and Zn (µg/kg).

1) δ^13^C_honey_

|  | **Mainland** | **Tasmania** | **Asia** | **Europe** |
| --- | --- | --- | --- | --- |
| **Tasmania** | 0.920 |  |  |  |
| **Asia** | 0.972 | 0.999 |  |  |
| **Europe** | 0.960 | 0.998 | >0.999 |  |
| **North America** | 0.707 | 0.443 | 0.510 | 0.433 |

3) Al

|  | **Mainland** | **Tasmania** | **Asia** | **Europe** |
| --- | --- | --- | --- | --- |
| **Tasmania** | >0.999 |  |  |  |
| **Asia** | 0.667 | 0.880 |  |  |
| **Europe** | 1.000 | >0.999 | 0.818 |  |
| **North America** | 0.749 | 0.913 | >0.999 | 0.871 |

5) B

|  | **Mainland** | **Tasmania** | **Asia** | **Europe** |
| --- | --- | --- | --- | --- |
| **Tasmania** | 0.995 |  |  |  |
| **Asia** | 0.923 | 0.871 |  |  |
| **Europe** | 0.998 | >0.999 | 0.848 |  |
| **North America** | 0.170 | 0.239 | 0.718 | 0.143 |

7) Cu

|  | **Mainland** | **Tasmania** | **Asia** | **Europe** |
| --- | --- | --- | --- | --- |
| **Tasmania** | 0.884 |  |  |  |
| **Asia** | 0.912 | 1.000 |  |  |
| **Europe** | 0.425 | 0.227 | 0.201 |  |
| **North America** | 0.951 | 0.999 | >0.999 | 0.270 |

2) δ^13^C_protein_

|  | **Mainland** | **Tasmania** | **Asia** | **Europe** |
| --- | --- | --- | --- | --- |
| **Tasmania** | 0.576 |  |  |  |
| **Asia** | 0.477 | >0.999 |  |  |
| **Europe** | 0.475 | 1.000 | 1.000 |  |
| **North America** | 0.658 | 0.154 | 0.105 | 0.095 |

4) Ba

|  | **Mainland** | **Tasmania** | **Asia** | **Europe** |
| --- | --- | --- | --- | --- |
| **Tasmania** | 0.062 |  |  |  |
| **Asia** | 0.248 | 0.930 |  |  |
| **Europe** | **0.011*** | >0.999 | 0.924 |  |
| **North America** | **0.037*** | >0.999 | 0.930 | >0.999 |

6) Ca

|  | **Mainland** | **Tasmania** | **Asia** | **Europe** |
| --- | --- | --- | --- | --- |
| **Tasmania** | **< 0.001***** |  |  |  |
| **Asia** | **< 0.001***** | 0.999 |  |  |
| **Europe** | 0.062 | 0.292 | 0.331 |  |
| **North America** | **0.007**** | 0.918 | 0.968 | 0.784 |

8) Fe

|  | **Mainland** | **Tasmania** | **Asia** | **Europe** |
| --- | --- | --- | --- | --- |
| **Tasmania** | 0.962 |  |  |  |
| **Asia** | **0.001**** | **0.004**** |  |  |
| **Europe** | >0.999 | 0.978 | **0.003**** |  |
| **North America** | 0.343 | 0.268 | 0.426 | 0.389 |

9) Mg

|  | **Mainland** | **Tasmania** | **Asia** | **Europe** |
| --- | --- | --- | --- | --- |
| **Tasmania** | **0.049*** |  |  |  |
| **Asia** | 0.249 | 0.904 |  |  |
| **Europe** | 0.989 | 0.160 | 0.556 |  |
| **North America** | 0.147 | 0.976 | 0.998 | 0.387 |

11) Ni

|  | **Mainland** | **Tasmania** | **Asia** | **Europe** |
| --- | --- | --- | --- | --- |
| **Tasmania** | 1.000 |  |  |  |
| **Asia** | 1.000 | 0.999 |  |  |
| **Europe** | 0.086 | 0.270 | 0.311 |  |
| **North America** | 0.998 | >0.999 | 0.994 | 0.156 |

13) K

|  | **Mainland** | **Tasmania** | **Asia** | **Europe** |
| --- | --- | --- | --- | --- |
| **Tasmania** | 0.972 |  |  |  |
| **Asia** | 0.447 | 0.368 |  |  |
| **Europe** | 0.786 | 0.999 | 0.110 |  |
| **North America** | 0.295 | 0.256 | 0.999 | 0.064 |

15) Na

|  | **Mainland** | **Tasmania** | **Asia** | **Europe** |
| --- | --- | --- | --- | --- |
| **Tasmania** | 0.263 |  |  |  |
| **Asia** | 0.932 | 0.769 |  |  |
| **Europe** | **0.042*** | 1.000 | 0.506 |  |
| **North America** | 0.137 | >0.999 | 0.649 | >0.999 |

17) Sn

|  | **Mainland** | **Tasmania** | **Asia** | **Europe** |
| --- | --- | --- | --- | --- |
| **Tasmania** | >0.999 |  |  |  |
| **Asia** | 0.368 | 0.669 |  |  |
| **Europe** | >0.999 | >0.999 | 0.413 |  |
| **North America** | 0.846 | 0.948 | 0.970 | 0.851 |

10) Mn

|  | **Mainland** | **Tasmania** | **Asia** | **Europe** |
| --- | --- | --- | --- | --- |
| **Tasmania** | 0.718 |  |  |  |
| **Asia** | **< 0.001***** | 0.129 |  |  |
| **Europe** | **0.020*** | 0.805 | 0.462 |  |
| **North America** | **< 0.001***** | 0.128 | >0.999 | 0.454 |

12) P

|  | **Mainland** | **Tasmania** | **Asia** | **Europe** |
| --- | --- | --- | --- | --- |
| **Tasmania** | 0.825 |  |  |  |
| **Asia** | 0.836 | >0.999 |  |  |
| **Europe** | **0.008**** | **0.008**** | **0.003**** |  |
| **North America** | 0.999 | 0.809 | 0.827 | 0.105 |

14) Rb

|  | **Mainland** | **Tasmania** | **Asia** | **Europe** |
| --- | --- | --- | --- | --- |
| **Tasmania** | 0.986 |  |  |  |
| **Asia** | 0.464 | 0.430 |  |  |
| **Europe** | 0.999 | 0.999 | 0.410 |  |
| **North America** | 0.618 | 0.541 | 1.000 | 0.548 |

16) Sr

|  | **Mainland** | **Tasmania** | **Asia** | **Europe** |
| --- | --- | --- | --- | --- |
| **Tasmania** | **0.014*** |  |  |  |
| **Asia** | **0.024*** | 0.986 |  |  |
| **Europe** | **0.007**** | 0.984 | >0.999 |  |
| **North America** | **0.014*** | 0.999 | 0.999 | 0.999 |

18) Zn

|  | **Mainland** | **Tasmania** | **Asia** | **Europe** |
| --- | --- | --- | --- | --- |
| **Tasmania** | 0.900 |  |  |  |
| **Asia** | 0.999 | 0.975 |  |  |
| **Europe** | 0.968 | 0.996 | 0.998 |  |
| **North America** | >0.999 | 0.928 | 0.999 | 0.981 |

Trace element concentrations with significant differences are plotted in Figure 2 of the main study.

**Supplementary Table S4.** Classification results from canonical discriminant analysis (CDA) of authentic commercial honey samples (n = 69) comprising mainland Australia (n = 24), Tasmania (n = 7) and overseas honey (n = 35) from Africa (n = 1), Asia (n = 10), Europe (n = 15) and North America (n = 9) in addition to honey samples of unknown origin (n = 3).

| **Geographic origins** | | | **Predicated group membership** | | | **Total** |
| --- | --- | --- | --- | --- | --- | --- |
|  |  |  | **Mainland** | **Overseas** | **Tasmania** |  |
| **Original** | **Count** | Mainland | 20 | 2 | 2 | 24 |
|  |  | Overseas | 2 | 31 | 2 | 35 |
|  |  | Tasmania | 0 | 2 | 5 | 7 |
|  |  | Unknown origin | 1 | 2 | 0 | 3 |
|  | **%** | Mainland | **83.3** | 8.3 | 8.3 | 100 |
|  |  | Overseas | 5.7 | **88.6** | 5.7 | 100 |
|  |  | Tasmania | 0 | 28.6 | **71.4** | 100 |
|  |  | Unknown origin | 33.3 | 66.7 | 0 | 100 |
| **Cross-validated ^a^** | **Count** | Mainland | 17 | 5 | 2 | 24 |
|  |  | Overseas | 2 | 29 | 4 | 35 |
|  |  | Tasmania | 1 | 2 | 4 | 7 |
|  | **%** | Mainland | **70.8** | 20.8 | 8.3 | 100 |
|  |  | Overseas | 5.7 | **82.9** | 11.4 | 100 |
|  |  | Tasmania | 14.3 | 28.6 | **57.1** | 100 |

CDA analysis resulted in 84.8% of original grouped cases correctly classified and 75.8% of cross-validated grouped cases correctly classified.

^a^ Cross validation was completed only for those cases in the analysis. In cross validation, each case was classified by the functions derived from all cases other than that case.

**Supplementary Table S5**. Loading values of the principal component analysis (PCA) presented in Figures 3a and 3b, respectively. Variables with loading values > 0.600 displayed in the tables are considered as the most important parameters for each component.

| **Rotated Component Matrix for Figure 3a** | | | | | | | | | | | | |
| --- | --- | --- | --- | --- | --- | --- | --- | --- | --- | --- | --- | --- |
| **Variables** | **Components** | | | | | | | | | | | |
|  | **1** | | **2** | | **3** | | **4** | | **5** | | **6** | |
| **δ^13^_honey_** |  | |  | |  | | 0.895 | |  | |  | |
| **δ^13^_protein_** |  | |  | |  | | 0.903 | |  | |  | |
| **Al** |  | |  | |  | |  | | 0.700 | |  | |
| **Ba** |  | | 0.716 | |  | |  | |  | |  | |
| **B** |  | |  | |  | |  | |  | | -0.746 | |
| **Ca** |  | | 0.849 | |  | |  | |  | |  | |
| **Cu** | 0.864 | |  | |  | |  | |  | |  | |
| **Fe** |  | |  | | 0.729 | |  | |  | |  | |
| **Mg** |  | | 0.629 | |  | |  | |  | |  | |
| **Mn** |  | | 0.628 | |  | |  | |  | |  | |
| **Ni** | 0.887 | |  | |  | |  | |  | |  | |
| **P** | 0.895 | |  | |  | |  | |  | |  | |
| **K** |  | |  | |  | |  | |  | |  | |
| **Rb** | 0.719 | |  | |  | |  | |  | |  | |
| **Na** |  | |  | | 0.655 | |  | |  | |  | |
| **Sr** |  | |  | |  | |  | | 0.841 | |  | |
| **Sn** |  | |  | | 0.836 | |  | |  | |  | |
| **Zn** |  | |  | |  | |  | |  | |  | |
| **Rotated Component Matrix for Figure 3b** | | | | | | | | | | | |  |
| **Variables** | **Components** | | | | | | | | | | |  |
|  | **1** | **2** | | **3** | | **4** | | **5** | | **6** | |  |
| **δ^13^_honey_** | -0.901 |  | |  | |  | |  | |  | |  |
| **δ^13^_protein_** | -0.716 |  | |  | |  | |  | |  | |  |
| **Al** |  |  | |  | | 0.641 | |  | |  | |  |
| **Ba** |  |  | |  | |  | | 0.792 | |  | |  |
| **B** | 0.914 |  | |  | |  | |  | |  | |  |
| **Ca** |  | 0.911 | |  | |  | |  | |  | |  |
| **Cu** |  |  | |  | |  | |  | | 0.848 | |  |
| **Fe** |  |  | | 0.692 | |  | |  | |  | |  |
| **Mg** |  | 0.857 | |  | |  | |  | |  | |  |
| **Mn** |  |  | |  | |  | | 0.716 | |  | |  |
| **Ni** |  |  | | 0.838 | |  | |  | |  | |  |
| **P** |  |  | |  | |  | | 0.765 | |  | |  |
| **K** |  |  | |  | | 0.894 | |  | |  | |  |
| **Rb** |  |  | |  | | 0.823 | |  | |  | |  |
| **Na** |  | 0.789 | |  | |  | |  | |  | |  |
| **Sr** |  |  | |  | |  | |  | |  | |  |
| **Sn** |  |  | |  | |  | |  | |  | |  |
| **Zn** |  |  | | 0.814 | |  | |  | |  | |  |

**Supplementary Table S6**. Loading values of the CDA for Figures 3c and 3d, respectively.

| **Structure Matrix for Figure 3c** | | |
| --- | --- | --- |
| **Variables** | **Functions** | |
|  | **1** | **2** |
| **Mn** | 0.475* | -0.243 |
| **Sr** | 0.417* | 0.284 |
| **Ba^b^** | 0.399* | 0.099 |
| **Ni^b^** | -0.232* | -0.065 |
| **Fe^b^** | 0.159* | 0.026 |
| **Zn^b^** | -0.130* | 0.108 |
| **Na^b^** | 0.121 | 0.017 |
| **Al^b^** | 0.073* | -0.065 |
| **Ca** | 0.421 | 0.444* |
| **B^b^** | 0.004 | 0.366* |
| **P** | -0.136 | 0.359* |
| **Mg^b^** | 0.215 | 0.299* |
| **Cu^b^** | -0.072 | 0.256* |
| **K** | 0.066 | -0.204* |
| **δ^13^_honey_^b^** | 0.046 | -0.101* |
| **δ^13^_protein_^b^** | 0.010 | 0.099* |
| **Rb^b^** | 0.068 | -0.082* |
| **Sn^b^** | 0.036 | -0.076* |

| **Structure Matrix for Figure 3d** | | |
| --- | --- | --- |
| **Variables** | **Functions** | |
|  | **1** | **2** |
| **Fe** | 0.291* | -0.216 |
| **Na** | 0.181* | -0.019 |
| **Ca** | -0.164* | 0.163 |
| **Sn** | 0.120* | -0.112 |
| **Ba** | 0.110* | 0.004 |
| **δ^13^_protein_** | 0.155 | 0.335* |
| **K** | -0.135 | 0.329* |
| **B** | -0.002 | -0.302* |
| **P** | -0.265 | 0.297* |
| **Mn** | -0.133 | 0.280* |
| **Cu** | -0.141 | 0.257* |
| **Ni** | -0.087 | 0.251* |
| **Mg** | -0.082 | 0.233* |
| **δ^13^_honey_** | 0.092 | 0.227* |
| **Rb** | -0.123 | 0.204* |
| **Al** | -0.105 | 0.182* |
| **Zn** | 0.016 | -0.180* |
| **Sr** | 0.032 | 0.072* |

| Pooled within-groups correlations between discriminating variables and standardized canonical discriminant functions.  Variables ordered by absolute size of the correlation within each function. |
| --- |
| * Largest absolute correlation between each variable and any discriminant function  ^b^ Variable not used in the analysis. |

**Supplementary Figure S1.** Concentrations (µg/kg) of trace elements Ca, Mn, Sr and P in authentic honey samples (n = 69) from mainland Australia (n = 24), Tasmania (n = 7), Africa (n = 1), Asia (n = 10), Europe (n = 15), North America (n = 9), and three unknown samples.

Trace elements Ca, Mn, Sr and P that had highly significant differences (*p* = <0.001–0.024; 5% significance) in Australian and international honeys (Supplementary Table S3) were selected as examples for scatterplots to show that there was no clear separate clustering according to the different regional and continental origin of honeys. Other paired trace elements (e.g. Ba, Fe, Mg, and Na) also had no clear geographic clustering. Combining any two of these elements did not separate honey according to its geographic origin. Therefore, PCA and CDA multivariate analysis was applied to all of the available variables (carbon isotopic ratios of honey and its protein and 16 trace elements). This approach resulted in clear groupings of honey derived from different geographic locations.

**Supplementary Figure S2**. Summary results of the C5.0 model for classification of the training set of authentic honey samples of known origin from mainland Australia, Tasmania, Asia, Europe and North America. The honey samples from Africa and those of unknown origin were excluded from the analysis.


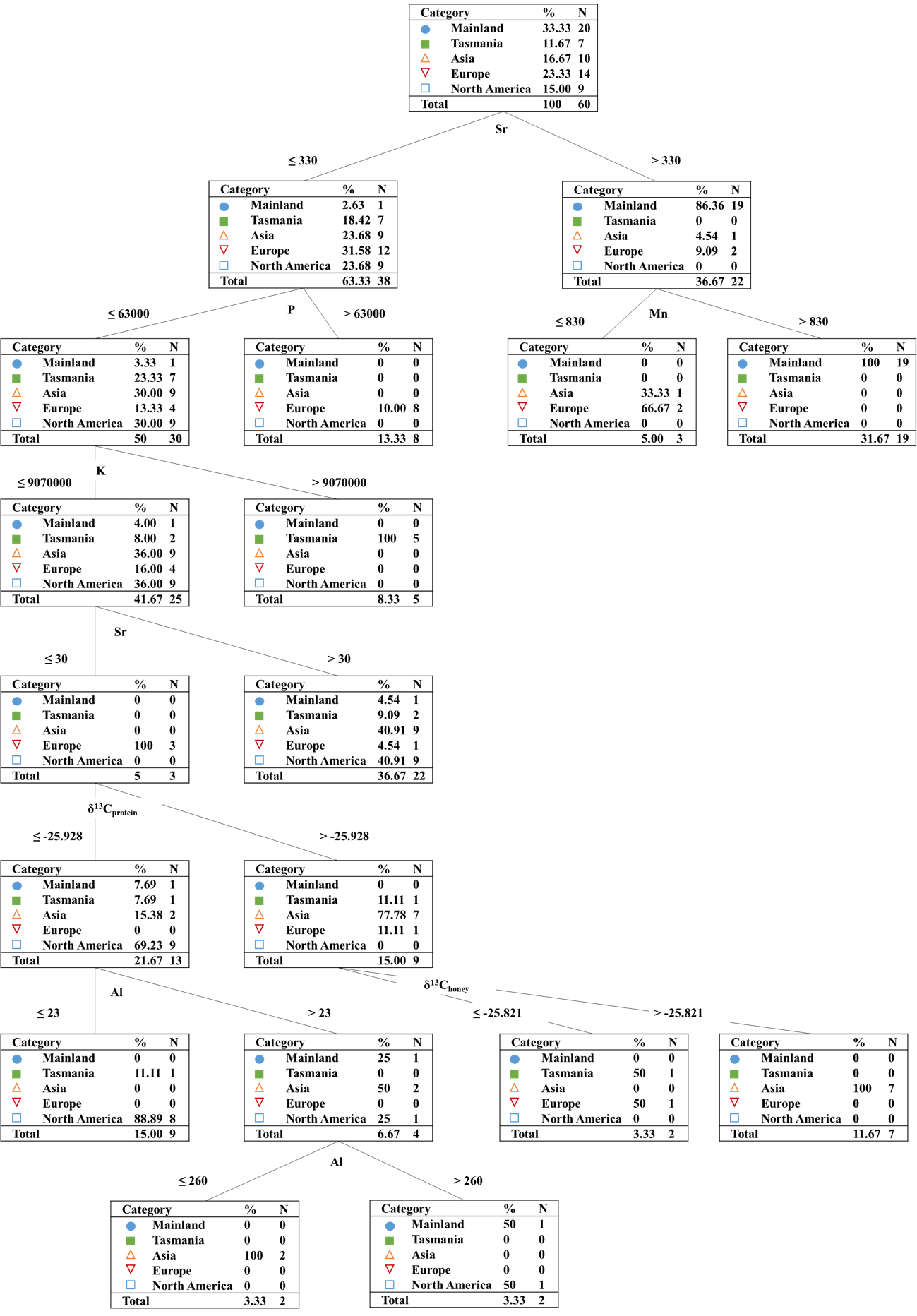


**Supplementary Figure S3.** Summary results of this study’s global investigation of honey authentication based upon analysis of honey δ^13^C, its protein and C-4 sugar content. Although the single African honey (n = 1) sample is excluded from the map it passed the C-4 criteria and is classified as an authentic honey.


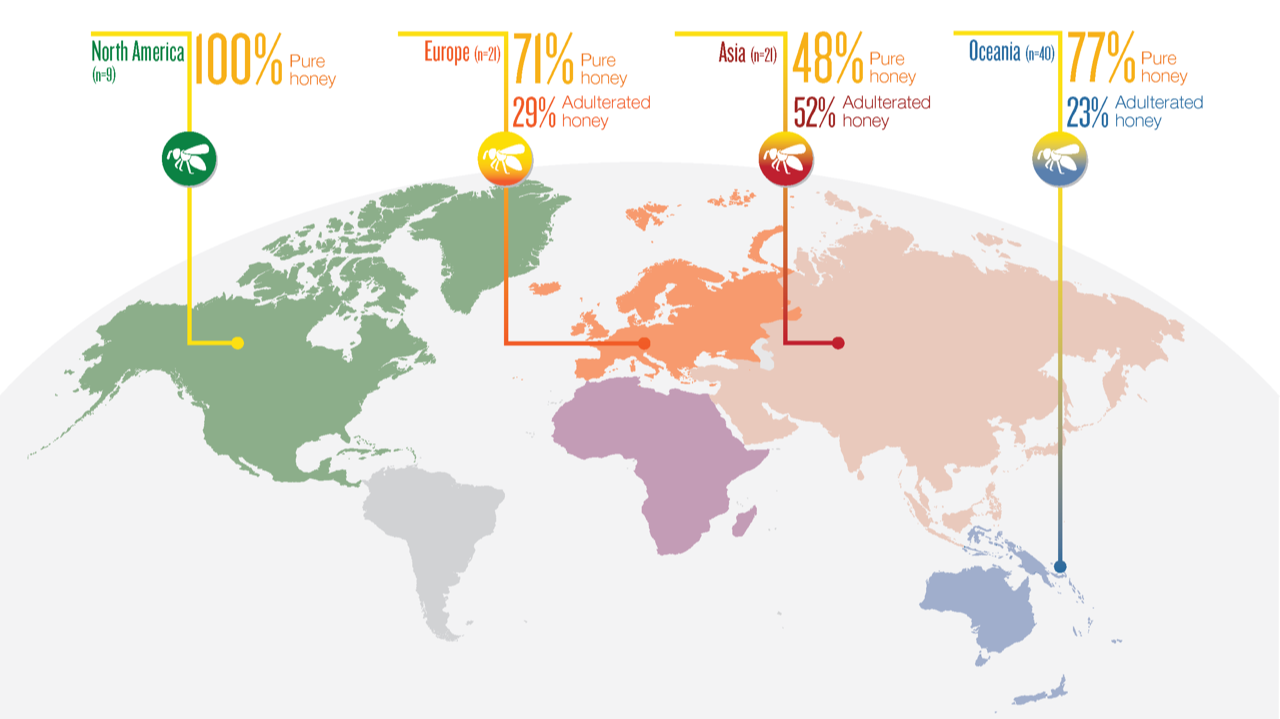


**References**

1 Association of Analytical Communities. AOAC Official Method 978.17. Corn and cane sugar products in honey. 27-29 (Arlington, 1995).

2 Padovan, G. J., Jong, D. D., Rodrigues, L. P. & Marchini, J. S. Detection of adulteration of commercial honey samples by the ^13^C/^12^C isotopic ratio. *Food Chem.* **82**, 633-636 (2003).

3 White, J. W. & Winters, K. Honey protein as internal standard for stable carbon isotope ratio detection of adulteration of honey. *J. Assoc. Off. Anal. Chem.* **72**, 907-911 (1989).

4 Simsek, A., Bilsel, M. & Goren, A. C. ^13^C/^12^C pattern of honey from Turkey and determination of adulteration in commercially available honey samples using EA-IRMS. *Food Chem.* **130**, 1115-1121 (2012).

5 Tosun, M. Detection of adulteration in honey samples added various sugar syrups with ^13^C/^12^C isotope ratio analysis method. *Food Chem.* **138**, 1629-1632 (2013).

6 Guler, A. *et al.* Detection of adulterated honey produced by honeybee (*Apis mellifera L.)* colonies fed with different levels of commercial industrial sugar (C(3) and C(4) plants) syrups by the carbon isotope ratio analysis. *Food Chem.* **155**, 155-160 (2014).

7 Elflein, L. & Raezke, K.-P. Improved detection of honey adulteration by measuring differences between ^13^C/^12^C stable carbon isotope ratios of protein and sugar compounds with a combination of elemental analyzer - isotope ratio mass spectrometry and liquid chromatography - isotope ratio mass spectrometry (δ^13^C-EA/LC-IRMS). *Apidologie* **39**, 574-587 (2008).

8 Association of Analytical Communities. AOAC Official Method 998.12. C4 plant sugars in honey. Internal standard stable carbon isotope ratio. 27-30 (Gaithersburg, 2014).

9 Dong, H. *et al.* Adulteration identification of commercial honey with the C-4 sugar content of negative values by an elemental analyzer and liquid chromatography coupled to isotope ratio mass spectroscopy. *J. Agric. Food Chem.* **64**, 3258-3265 (2016).
